# DNA Barcode library of the endemic-rich avifauna of the oceanic islands of the Gulf of Guinea

**DOI:** 10.3897/BDJ.11.e110428

**Published:** 2023-10-23

**Authors:** Martim Melo, Rita Covas, Ricardo Faustino de Lima, Octávio Veiga da Horta, Ceciliano do Bom Jesus, Martim Barros da Veiga, Seduney Samba, Ricardo Fonseca, Gabriel Cabinda, Lionel Viegas, Teresa L Silva, Vanessa A. Mata, Pedro Beja, Sónia Ferreira

**Affiliations:** 1 CIBIO, Centro de Investigação em Biodiversidade e Recursos Genéticos, InBIO Laboratório Associado, Campus de Vairao, Universidade do Porto, 4485-661 Vairao, Vila do Conde, Portugal CIBIO, Centro de Investigação em Biodiversidade e Recursos Genéticos, InBIO Laboratório Associado, Campus de Vairao, Universidade do Porto, 4485-661 Vairao Vila do Conde Portugal; 2 BIOPOLIS Program in Genomics, Biodiversity and Land Planning, CIBIO, Campus de Vairao, 4485-661 Vairao, Vila do Conde, Portugal BIOPOLIS Program in Genomics, Biodiversity and Land Planning, CIBIO, Campus de Vairao, 4485-661 Vairao Vila do Conde Portugal; 3 MHNC-UP, Museu de História Natural e da Ciência da Universidade do Porto, Porto, Portugal MHNC-UP, Museu de História Natural e da Ciência da Universidade do Porto Porto Portugal; 4 DST/NRF Centre of Excellence, FitzPatrick Institute, University of Cape Town, Rondebosch, South Africa DST/NRF Centre of Excellence, FitzPatrick Institute, University of Cape Town Rondebosch South Africa; 5 Gulf of Guinea Biodiversity Centre, São Tomé, São Tomé and Príncipe Gulf of Guinea Biodiversity Centre São Tomé São Tomé and Príncipe; 6 Centre for Ecology, Evolution and Environmental Changes, Faculdade de Ciências da Universidade de Lisboa, Lisboa, Portugal Centre for Ecology, Evolution and Environmental Changes, Faculdade de Ciências da Universidade de Lisboa Lisboa Portugal; 7 Departamento de Biologia Animal, Faculdade de Ciências, Universidade de Lisboa, Lisboa, Portugal Departamento de Biologia Animal, Faculdade de Ciências, Universidade de Lisboa Lisboa Portugal; 8 CHANGE - Global Change and Sustainability Institute, Faculdade de Ciências, Universidade de Lisboa, Lisboa, Portugal CHANGE - Global Change and Sustainability Institute, Faculdade de Ciências, Universidade de Lisboa Lisboa Portugal; 9 Associação Monte Pico, Monte Café, São Tomé, São Tomé and Príncipe Associação Monte Pico, Monte Café São Tomé São Tomé and Príncipe; 10 Parque Natural do Obô – Príncipe, Porto Real, Príncipe, São Tomé and Príncipe Parque Natural do Obô – Príncipe, Porto Real Príncipe São Tomé and Príncipe; 11 Parque Nacional do Obô - São Tomé, São Tomé, São Tomé and Príncipe Parque Nacional do Obô - São Tomé São Tomé São Tomé and Príncipe; 12 CIBIO, Centro de Investigação em Biodiversidade e Recursos Genéticos, InBIO Laboratório Associado, Instituto Superior de Agronomia, Universidade de Lisboa, Lisboa, Portugal CIBIO, Centro de Investigação em Biodiversidade e Recursos Genéticos, InBIO Laboratório Associado, Instituto Superior de Agronomia, Universidade de Lisboa Lisboa Portugal

**Keywords:** Annobón, aves, COI, DNA barcode, São Tomé, Príncipe

## Abstract

**Background:**

The BioSTP: DNA Barcoding of endemic birds from oceanic islands of the Gulf of Guinea dataset contains records of 155 bird specimens belonging to 56 species in 23 families, representing over 80% of the diversity of the breeding landbird community. All specimens were collected on Príncipe, São Tomé and Annobón Islands between 2002 and 2021 and morphologically identified to species or subspecies level by qualified ornithologists. The dataset includes all endemic species and 3/4 of the extant endemic subspecies of the islands. This dataset is the second release by BioSTP and it greatly increases the knowledge on the DNA barcodes of Gulf of Guinea birds. All DNA extractions are deposited at Associação BIOPOLIS - CIBIO, Research Center in Biodiversity and Genetic Resources.

**New information:**

The dataset includes DNA barcodes for all 29 endemic bird species and for 11 of the 15 extant endemic bird subspecies from the oceanic islands of the Gulf of Guinea. This is the first major DNA barcode set of African birds. The three endemic subspecies of *Crithagrarufobrunnea*, an island endemic with three allopatric populations within the Archipelago, are also represented. Additionally, we obtained DNA barcodes for 16 of the 21 non-endemic landbirds and for one vagrant (*Sylviacommunis*). In total, forty-one taxa were new additions to the Barcode of Life Data System (BOLD), with another 11 corresponding to under-represented taxa in BOLD. Furthermore, the submitted sequences were found to cluster in 55 Barcode Index Numbers (BINs), 37 of which were new to BOLD. All specimens have their DNA barcodes publicly accessible through BOLD online database and GenBank.

## Introduction

**Figure 1.**
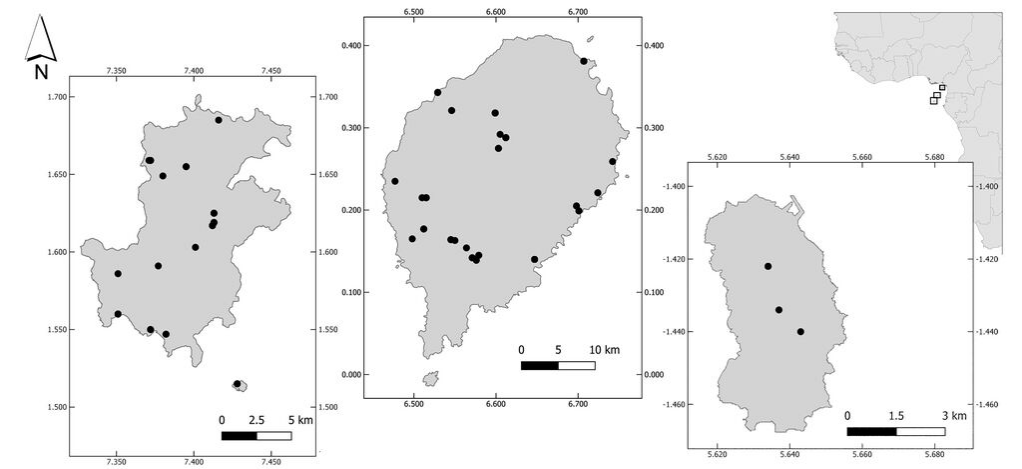
Map of the localities where birds samples were collected in the oceanic islands of the Gulf of Guinea: Principe (left), São Tomé (centre) and Annobón (right). Top right: location of the Gulf of Guinea islands in Africa.

The Gulf of Guinea islands, off the Atlantic coast of central Africa, form the offshore part of the Cameroon Line of Volcanoes, which stretches 1,600 km from Annobón, in the SW, to the Mandara Mountains, in the NE (Ceríaco et al. 2022a). They include three oceanic islands – Príncipe, São Tomé and Annobón – and the land-bridge Island of Bioko (Fig. 1). Príncipe and São Tomé form the Democratic Republic of São Tomé and Príncipe, whereas Annobón and Bioko are part of the Republic of Equatorial Guinea.

The three oceanic islands lie in seas over 1,800 m deep and were never connected to each other or to the mainland (Ceríaco et al. 2022a). The origin of Príncipe, ca. 31 Ma, dates back to the origin of the Cameroon line; the origin of São Tomé is estimated at 15 Ma and that of Annobón at 6 Ma. However, due to a long history of intense volcanic activity, the oldest sub-aerial rocks may have been covered by subsequent lava flows. Volcanic activity was present until as recently as 36 Ka (Barfod and Fitton 2014).

The islands share an oceanic equatorial climate with extensive rainy seasons and very high humidity throughout most of the year (Ceríaco et al. 2022a). On Príncipe and São Tomé, there is a main dry season from mid-May to late August and a short dry season that lasts for a few weeks, sometime between December and February. On Annobón, south of the Equator, there is a single, but longer dry season, from mid-May to the end of October. The predominant warm and moist SW winds are intercepted by the high relief of the islands, creating a north-south divide in annual precipitation that, in São Tomé, can go from over 7,000 mm in the southwest to less than 600 mm in the north. Mean annual temperatures are above 25ºC at sea level, decreasing with altitude, where mean maximum temperatures can be similar, but the absolute minimums are much lower, falling below 10ºC at 700 m a.s.l.

The three oceanic islands were uninhabited at the time of discovery by the Portuguese in the late 15^th^ century (Muñoz-Torrent et al. 2022). The Gulf of Guinea islands were then almost entirely covered by tropical moist broadleaf forests (Dauby et al. 2022), which are stratified by altitude into lowland, montane and mist forests. The latter type is absent from Príncipe. In Annobón, these altitudinal belts are compressed in an altitudinal range that only goes up to 700 m and a similar phenomenon seems to occur in the southern peaks of São Tomé (Ogonovszky 2003). On São Tomé, species such as *Ericathomensis* (Ericaceae) and *Lobeliabarnsii* (Campanulaceae), found in clearings close to the highest peak, are representatives of an incipient montane grassland (Figueiredo et al. 2011). On São Tomé and on Annobón, the strong rain-shadow effect on the north creates a distinct dry forest, currently highly altered by human action (Diniz and Matos 2002, Lebigre 2003). The original vegetation also includes coastal sand dunes communities and mangroves, even though these have always covered tiny portions of the islands (Diniz and Matos 2002, Dauby et al. 2022).

The three oceanic islands of the Gulf of Guinea host an outstanding high number of endemic species across taxonomic groups (Ceríaco et al. 2022b, Melo et al. 2022a). Their avifauna is particularly unique. With at least 29 endemic bird species in an area of just over 1,000 km^2^, these oceanic islands have the highest concentration of endemic birds in the world (Melo et al. 2022b). Many of the endemics are threatened (IUCN 2023), making the islands a top global priority for conservation. The south-western forests of São Tomé ranked as the second most important for bird conservation in Africa (Collar and Stuart 1988) and, more recently, the moist lowland forests of the three islands were considered the third most important ecoregion for the conservation of forest birds globally (Buchanan et al. 2011). The international recognition of the global relevance of these islands for biodiversity has led to a steady build-up of conservation efforts, which nevertheless remain insufficient (de Lima et al. 2022). Each island has its own protected area: Príncipe Obô Natural Park, São Tomé Obô Natural Park (both since 2006) and Annobón Nature Reserve (since 2000). Additionally, Príncipe was declared a UNESCO Biosphere Reserve in 2012. In Príncipe and in São Tomé, the parks cover around one third of each island, including most of the remaining native forest, whereas Annobón’s protected area covers the whole island. In a global analysis including more than 175,000 protected areas, the natural park of São Tomé came up as the 17^th^ most irreplaceable area for the conservation of threatened vertebrates, whereas the small natural park of Príncipe came in the 265^th^ place (Le Saout et al. 2013). The diversity and threat levels of the endemic birds were a major factor behind these high rankings.

The avifauna of the oceanic islands of the Gulf of Guinea comprises 148 confirmed species, of which 66 are resident, six are breeding seabirds and two feral species (de Lima and Melo 2021). The breeding avian community is characterised by high phylogenetic diversity, with 33 families represented. Endemism is restricted to the resident landbirds, which include 29 endemic species and 16 subspecies from species with mainland populations, most of which are restricted to a single island. This includes the endemic subspecies of the Olive Ibis *Bostrychiaolivacea* from Príncipe (spp. *rothschildi*) that became extinct during the 20^th^ century (de Lima and Melo 2021). Additionally, the endemic Principe Seedeater *Crithagrarufobrunnea* has diversified into three endemic subspecies within the Archipelago. Excluding the 15 resident species that are likely non-native, 82% of the confirmed resident native birds are endemic taxa.

The striking scarcity of genetic data associated with the high biodiversity found in São Tomé and Príncipe instigated the creation of a DNA barcoding initiative by the Research Network in Biodiversity and Evolutionary Biology - InBIO (Associate Laboratory). DNA barcoding is a method that aims to identify organisms, based on a short DNA sequence previously sequenced from morphologically identified specimens (Herbert et al. 2003). This requires the construction of comprehensive reference collections of DNA sequences that represent the existing biodiversity (Kress et al. 2005, Baird et al. 2011, Ferreira et al. 2018). DNA barcoding can also be used as a first step in new species discovery and, as such, can be used as a tool to help address the taxonomic impediment problem (e.g. Kekkonen and Hebert (2014)). The BioSTP: DNA Barcoding for São Tomé and Princípe makes use of Next Generation Sequencing technologies (NGS) to develop a reference collection of DNA barcoding sequences. The first DNA barcode library has just been released for all amphibians and reptiles of São Tomé and Príncipe (Ceríaco et al. 2023). The bird dataset presented here is the first to cover taxa of the three oceanic islands of the Gulf of Guinea.

The DS-IAAST BioSTP: DNA Barcoding of endemic birds from oceanic islands of the Gulf of Guinea dataset contains records of 155 specimens of birds collected in São Tomé, Principe and Annobon islands, all of which were morphologically identified to species or suspecies level, for a total of 56 species. All specimens have their DNA barcodes made publicly available in the Barcode of Life Data System (BOLD) and GenBank. We have included in this dataset the barcodes of all identified birds specimens in DS-IAAST up to October 2021. Overall, this paper is a contribution to sharing and publicly disseminating the DNA barcodes of specimens from our reference collection to increase the available information on the Gulf of Guinea bird fauna.

## General description

### Purpose

This dataset aims to provide a contribution to the knowledge on the DNA barcode sequence library for the endemic birds of the oceanic islands of the Gulf of Guinea. Such a library should facilitate DNA-based identification of species for both traditional molecular studies and DNA metabarcoding studies and constitute a valuable resource for taxonomic and ecological research in the region.

### Additional information

We obtained the full DNA barcode sequence of cytochrome c oxidase I (COI – 658 bp Folmer region) for 155 specimens (Table 1, Fig. 1). Fig. 2 and Fig. 3 illustrate examples of the diversity of species that are part of the dataset. Sequences are distributed in 55 Barcode Index Numbers (BINs), 37 of which are unique to this dataset. Genetic distances between species of the same genus vary from 0.46% between *Crithagrarufobrunnea* and *Crithagraconcolor* (two endemics) and 15.62% between *Columbamalherbii* and *Columbalarvata*.

Despite sharing the same BINs, DNA barcoding seems also to be useful to distinguish the pairs of the closely-related sister species *Zosteropsleucophaeus* (Principe) and *Zosteropslugubris* (São Tomé), *Lamprotornisornatus* (Principe) and *Lamprotornissplendidus* (Principe) and the pairs of subspecies *Corythorniscristatusnais* (Principe) and *Corythorniscristatusthomensis* (São Tomé), despite the reduced genetic distances observed (p-distances = 0.61%, 0.77% and 0.46%, respectively), as they exhibited consistent differences. In contrast, the three subspecies of *Crithagrarufobrunnea* could not be identified using DNA barcoding, based on COI sequences. Nevertheless, there is evidence that justifies their current treatment as distinct subspecies, based on the reduced gene flow between the three allopatric populations and observable phenotypic differentiation (Melo 2007, Melo et al. 2022b). In the case of *Columbalarvata* subspecies, DNA barcodes of two of the three subspecies were sequenced and, although two distinct BINs were attributed, it seems not possible to distinguish *C.l.principalis* from *C.l.simplex* specimens.

Despite repeated attempts, we failed to obtain the full DNA barcode sequence for *Treronsanctithomae*, of which we could only obtain and sequence a smaller fragment: 325 bp using primers FwhF1 (Vamos et al. 2017) + C_R (Shokralla et al. 2015) for LC amplification. Likewise, no DNA barcode could be obtained from its sister species, *Treroncalvus*, which has an endemic subspecies on Principe. For this taxon, we only managed to obtain a sequence of a nuclear copy amplified with primers BF3 (Elbrecht et al. 2019) + BR2 (Elbrecht and Leese 2017), identified as such by the presence of a stop codon. Other researchers had previously generated DNA sequences of COI fragments of *T.calvus*: Pereira et al. (2007) (GenBank Accession: EF373392), Pereira (2013) (unpublished sequences) and Nowak et al. 2019 (GenBank Accession: KT023356), but no-one was able to generate a full DNA barcode so far and, therefore, no BIN has been attributed to this taxon.

## Project description

### Title

The name "BioSTP: DNA Barcoding of the endemic birds from oceanic islands of the Gulf of Guinea dataset" refers to data release of DNA barcodes and distribution data of birds in BOLD Systems.

### Personnel

Martim Melo (ornithologist), Rita Covas (ornithologist), Ricardo Faustino de Lima (ornithologist), Vanessa Mata (ecologist), Teresa Luisa Silva (project technician), Cátia Chaves (project technician), Joana Pinto (project technician), Octávio Veiga da Horta (field technician), Ceciliano do Bom Jesus (field technician), Martim Barros da Veiga (field technician), Seduney Samba (field technician), Ricardo Fonseca (field technician), Gabriel Cabinda (field technician), Pedro Beja (project coordinator) and Sónia Ferreira (DNA barcoding coordinator).

### Study area description

Oceanic islands of the Gulf of Guinea: Príncipe, São Tomé, Annobón

Príncipe Island is 139 km^2^ and lies 220 km west of the coast of Central Africa and 146 km northeast of São Tomé Island (Ceríaco et al. 2022a). It comprises a relatively flat, low-lying basalt platform in the north, contrasting with a rugged and mountainous south, where the main peaks are Pico do Príncipe (948 m a.s.l.), Mencorne (935 m a.s.l.) and Carriote (830 m a.s.l.). Once completely covered in rainforest, most accessible areas have been cleared and planted, even though some have regrown and are now covered with secondary forest (de Lima et al. 2022). The remaining native forest is mostly restricted to rugged terrain, including some lowland forest in the south and the montane forest around Pico do Príncipe.

São Tomé Island is 857 km^2^ and lies 255 km west of Gabon and 150 km southwest of Príncipe Island (Ceríaco et al. 2022a). The Equator passes through Ilhéu das Rolas, an islet just south of the main island. The Island is cone-shaped, typical of islands with recent events of volcanism. It reaches its highest point at Pico de São Tomé (2,024 m a.s.l.), even though there are many high peaks and volcanic plugs spread across the Island. Native vegetation is mostly restricted to the rugged centre and southwest (de Lima et al. 2022). Nevertheless, only a few areas have currently no forest cover, such as the fire-prone anthropogenic savannahs in the north, the few urban centres spread mostly along the coast and northeast, the horticultural areas at higher altitudes, coconut groves along the coast and oil palm monocultures in the south. Most agricultural areas correspond to agroforestry systems with dense canopy cover, such as cocoa and coffee shade plantations or forest gardens and there are also extensive portions of the island that are covered by secondary growth.

Annobón Island is 17 km^2^ and lies 340 km west of the mainland and 180 km southwest of São Tomé Island (Ceríaco et al. 2022a). The central part of the Island is comprised by the crater of Quioveo (640 m a.s.l.) and by Santamina, the highest point (700 m a.s.l.). Other geologic landmarks include Pico do Fogo, a trachyte plug (450 m a.s.l.) and Lago A Pot, a small crater lake (220 m a.s.l.). Only three valleys contain permanent streams and the north has savannah-like formations and dry bush, which are surrounded by dry lowland forest to the south (de Lima et al. 2022). The south of the Island is covered by taller mist-forest covered by epiphytes. The vegetation is reported to have been less modified by humans than on São Tomé and Príncipe and there is little sign of cocoa and coffee plantations, which are now abandoned and colonised by secondary alien-rich regrowth. Nevertheless, the north has been most affected and most flat areas up to the inside of the crater are cultivated.

### Design description

A total of 155 specimens were collected in the field, morphologically identified and DNA barcoded.

### Funding

The present study was funded by the project TROPIBIO NORTE-01-0145-FEDER-000046, supported by Norte Portugal Regional Operational Programme (NORTE2020), under the PORTUGAL 2020 Partnership Agreement, through the European Regional Development Fund (ERDF) and was carried out in collaboration with BirdLife International, in the framework of the partnership agreement between BirdLife International and BIOPOLIS association to promote informed, evidence-based biodiversity conservation action in São Tomé and Príncipe, which is funded by the European Union through the ‘Landscape Management in São Tomé and Príncipe’ project (ENV/2020/420-182), has received funding from the European Union’s Horizon 2020 Research and Innovation Programme under grant agreement No 854248 and by the project NORTE-01-0246-FEDER-000063, supported by Norte Portugal Regional Operational Programme (NORTE2020), under the PORTUGAL 2020 Partnership Agreement, through the European Regional Development Fund (ERDF). SF and VM were funded by the Fundação para a Ciência e a Tecnologia through the programme ‘Stimulus of Scientific Employment, Individual Support — 3rd Edition’ 2020.03526.CEECIND; 2020.02547.CEECIND). FCT provided structural funding to CIBIO-InBIO (UIDB/50027/2021) and cE3c (UID/BIA/00329/2023).

## Sampling methods

### Study extent

Oceanic islands of the Gulf of Guinea: São Tomé, Príncipe and Annobón

### Sampling description

Blood samples were collected non-destructively by puncturing the brachial vein of mist-netted birds, except for *Bostrychiabocagei* for which feathers were obtained from a live juvenile captured by a local hunter. Samples were preserved in 96% ethanol, which were kept at room temperature in the field and at -80ºC in the lab.

DNA was extracted using the QIAmp DNA Micro Kit that is designed to extract higher concentrations of genetic material from samples with small amounts of DNA. DNA amplification was performed using two different primer pairs, that amplify partially overlapping fragments (LC + BH) of the 658 bp barcoding region of the COI mitochondrial gene. We used the primers FwhF1 (Vamos et al. 2017) + C_R (Shokralla et al. 2015) for LC and BF3 (Elbrecht et al. 2019) + BR2 (Elbrecht and Leese 2017) for BH amplification, all modified with Illumina adaptors. PCRs were performed in 10 µl reactions, containing 5 µl of Multiplex PCR Master Mix (Qiagen, Germany), 0.3 µl of each 10 mM primer and 1-2 4 µl of DNA, with the remaining volume in water. PCR cycling conditions consisted of an initial denaturation at 95ºC for 15 min, followed by 45 cycles of denaturation at 95ºC for 30 sec, annealing at 45ºC for 45 sec and extension at 72ºC for 45 sec and a final elongation step at 60ºC for 10 min.

Successfull amplification was validated through 2% agarose gel electrophoresis and samples selected for sequencing followed for a second PCR, where Illumina P5 and P7 adapters with custom 7 bp long barcodes were attached to each PCR product. The index PCR was performed in a volume of 10 µl, including 5 µl of KAPA HiFi PCR Kit (KAPA Biosystems, U.S.A.), 0.5 µl of each 10 mM indexing primer and 2 µl of diluted PCR product (usually 1:4). PCR cycling conditions were as before, except that only 10 cycles were performed and at an annealing temperature of 55ºC. The amplicons were purified using AMPure XP beads (New England Biolabs, U.S.A.) and quantified using NanoDrop 1000 (Thermo Scientific, USA). Clean PCR products were then pooled equimolarly per fragment. Each pool was quantified with KAPA Library Quantification Kit Illumina® Platforms (KAPA Biosystems, USA) and the 2200 Tapestation System (Agilent Technologies, California, USA) was used for fragment length analysis prior to sequencing (Paupério et al. 2018). DNA sequencing was done at CIBIO facilities on an Illumina MiSeq benchtop system, using a V2 MiSeq sequencing kit (2 x 250 bp).

Illumina sequencing reads were processed using OBITools (Boyer et al. 2015) and VSEARCH (Rognes et al. 2016). Briefly, paired-end reads were aligned, collapsed into exact sequence variants, filtered by length, denoised and checked for chimeras. The resulting sequences from both LC and BH fragments of each sample were further assembled using CAP3 (Huang and Madan 1999) to produce a single 658 bp contig per sample.

### Quality control

All DNA barcodes sequences were compared against the BOLD database and the 99 top hits were inspected in order to detect possible issues due to contaminations or misidentifications.

### Step description

1. Blood samples were collected non-destructively by puncturing the brachial vein of mist-netted birds, except for *Bostrychiabocagei* for which feathers were obtained from a live juvenile captured by a local hunter.

2. Blood or tissue samples were preserved in 96% ethanol at room temperatur in the field and at -80ºC in the lab.

3. All specimens were morphologically identified and DNA barcoded. To sequence the 658 bp COI DNA barcode fragment, one leg was removed from each individual, DNA was extracted and then amplified. All DNA extracts were deposited in the DS-IAAST collection.

4. All sequences in the dataset were submitted to BOLD and GenBank database and, to each sequenced specimen, the morphological identification was contrasted with the results of the BLAST of the newly-generated DNA barcodes in the BOLD Identification Engine.

## Geographic coverage

### Description

Oceanic islands of the Gulf of Guinea: Príncipe, São Tomé and Annobón Islands

### Coordinates

S 1.475 and N 1.710 Latitude; E 5.634 and E 7.471 Longitude.

## Taxonomic coverage

### Description

This dataset is composed entirely of data relating to 155 birds records.

Overall, 60 taxa are represented in the dataset: 29 endemic species (one with three endemic subspecies), 11 endemic subspecies from taxa with mainland populations, four native residents, 11 probably non-native residents, two of uncertain status and one vagrant. These represent 56 species distributed in 23 families, over 80% of the diversity of the breeding land-bird community (de Lima and Melo 2021).

### Taxa included

**Table:** 

Rank	Scientific Name	Common Name
kingdom	Animalia	Animals
phylum	Chordata	Vertebrates
class	Aves	Birds
order	Apodiformes	
order	Columbiformes	
order	Coraciiformes	
order	Cuculiformes	
order	Galliformes	
order	Gruiformes	
order	Passeriformes	
order	Pelecaniformes	
order	Psittaciformes	
order	Strigiformes	
family	Alcedinidae	
family	Apodidae	
family	Ardeidae	
family	Cisticolidae	
family	Columbidae	
family	Cuculidae	
family	Estrildidae	
family	Fringillidae	
family	Laniidae	
family	Monarchidae	
family	Motacillidae	
family	Nectariniidae	
family	Oriolidae	
family	Phasianidae	
family	Ploceidae	
family	Psittacidae	
family	Rallidae	
family	Strigidae	
family	Sturnidae	
family	Sylviidae	
family	Threskiornithidae	
family	Turdidae	
family	Zosteropidae	

## Temporal coverage

**Data range:** 2002-12-05 – 2021-10-17.

### Notes

Samples were collected in the period from 05 December 2002 to 17 October 2021.

## Collection data

### Collection name

BioSTP: DNA Barcoding of endemic birds from oceanic islands of the Gulf of Guinea.

### Curatorial unit

DNA extractions - 1 to 156.

## Usage licence

### Usage licence

Creative Commons Public Domain Waiver (CC-Zero)

## Data resources

### Data package title

BioSTP: DNA Barcoding of endemic birds from oceanic islands of the Gulf of Guinea

### Number of data sets

1

### Data set 1.

#### Data set name

DS-IAAST BioSTP: endemic birds

#### Data format

dwc, xml, tsv, fasta

#### Download URL

https://dx.doi.org/10.5883/DS-IAAS T

#### Description

The BioSTP: DNA Barcoding of endemic birds from oceanic islands of the Gulf of Guinea dataset can be downloaded from the Public Data Portal of BOLD in different formats (data as dwc, xml or tsv and sequences as fasta files). Alternatively, BOLD users can log-in and access the dataset via the Workbench platform of BOLD. All records are also searchable within BOLD, using the search function of the database. The version of the dataset, at the time of writing the manuscript, is included as Suppl. materials 1, 2, 3. Column labels below follow the labels downloaded in the tsv format. Columns with no content in our dataset are left out in the list below.

**Data set 1.** 

Column label	Column description
processid	Unique identifier for the sample.
sampleid	Identifier for the sample being sequenced, i.e. DS-IAAST catalogue number at Cibio-InBIO, Porto University. Often identical to the "Field ID" or "Museum ID".
recordID	Identifier for specimen assigned in the field.
catalognum	Catalogue number.
fieldnum	Field number.
institution_storing	The full name of the institution that has physical possession of the voucher specimen.
bin_uri	Barcode Index Number system identifier.
phylum_taxID	Phylum taxonomic numeric code.
phylum_name	Phylum name.
class_taxID	Class taxonomic numeric code.
class_name	Class name.
order_taxID	Order taxonomic numeric code.
order_name	Order name.
family_taxID	Family taxonomic numeric code.
family_name	Family name.
subfamily_taxID	Subfamily taxonomic numeric code.
subfamily_name	Subfamily name.
genus_taxID	Genus taxonomic numeric code.
genus_name	Genus name.
species_taxID	Species taxonomic numeric code.
species_name	Species name.
identification_provided_by	Full name of primary individual who assigned the specimen to a taxonomic group.
identification_method	The method used to identify the specimen.
voucher_status	Status of the specimen in an accessioning process (BOLD controlled vocabulary).
tissue_type	A brief description of the type of tissue or material analysed.
collectors	The full or abbreviated names of the individuals or team responsible for collecting the sample in the field.
lifestage	The age class or life stage of the specimen at the time of sampling.
sex	The sex of the specimen.
lat	The geographical latitude (in decimal degrees) of the geographic centre of a location.
lon	The geographical longitude (in decimal degrees) of the geographic centre of a location.
elev	Elevation of sampling site (in metres above sea level).
country	The full, unabbreviated name of the country where the organism was collected.
province_state	The full, unabbreviated name of the province where the organism was collected.
region	The full, unabbreviated name of the municipality where the organism was collected.
exactsite	Additional name/text description regarding the exact location of the collection site relative to a geographic relevant landmark.

## Supplementary Material

68D0ABDC-2CB6-57BA-B119-152D06FEE838 10.3897/BDJ.11.e110428.suppl1 Supplementary material 1 DS-IAAST library - Specimen details Data type Record information - specimen data Brief description The file includes information about all records in BOLD for the DS-IAAST library. It contains collecting and identification data. The data are as downloaded from BOLD, in the tsv format, without further processing, except line endings were replaced with plain ones (linefeed only, LF). File: oo_886913.txt https://binary.pensoft.net/file/886913 Martim Melo, Vanessa A. Mata, Pedro Beja, Sónia Ferreira

5B287CD8-0D41-5190-A280-1789FB881CD2 10.3897/BDJ.11.e110428.suppl2 Supplementary material 2 DS-IAAST library Data type Specimen data records in Darwin Core Standard format Brief description The file includes information about all records in BOLD for the DS-IAAST library. It contains collecting and identification data. The data are in the DWC format. File: oo_887240.txt https://binary.pensoft.net/file/887240 Martim Melo, Vanessa A. Mata, Pedro Beja, Sónia Ferreira

B0EEDA28-2668-5AAF-B8DF-21DBAAA36CB5 10.3897/BDJ.11.e110428.suppl3 Supplementary material 3 DS-IAAST library - DNA sequences Data type Specimen genomic data, DNA sequences Brief description COI sequences in fasta format. Each sequence is identified by the BOLD ProcessID, species name, marker and GenBank accession number, separated by pipe. The data are as downloaded from BOLD, without further processing, except line endings were replaced with plain ones (linefeed only, LF). File: oo_886911.txt https://binary.pensoft.net/file/886911 Martim Melo, Vanessa A. Mata, Pedro Beja, Sónia Ferreira

## Figures and Tables

**Figure 2a.**
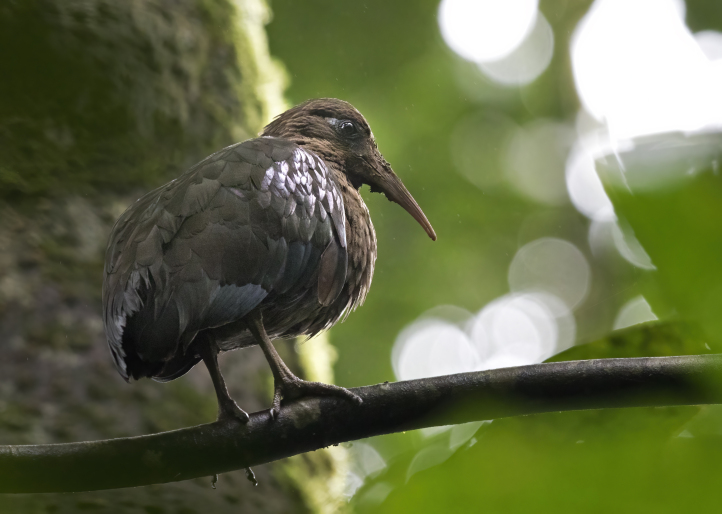
São Tomé Ibis *Bostrychiabocagei*. BIN URI BOLD:AEW0523.

**Figure 2b.**
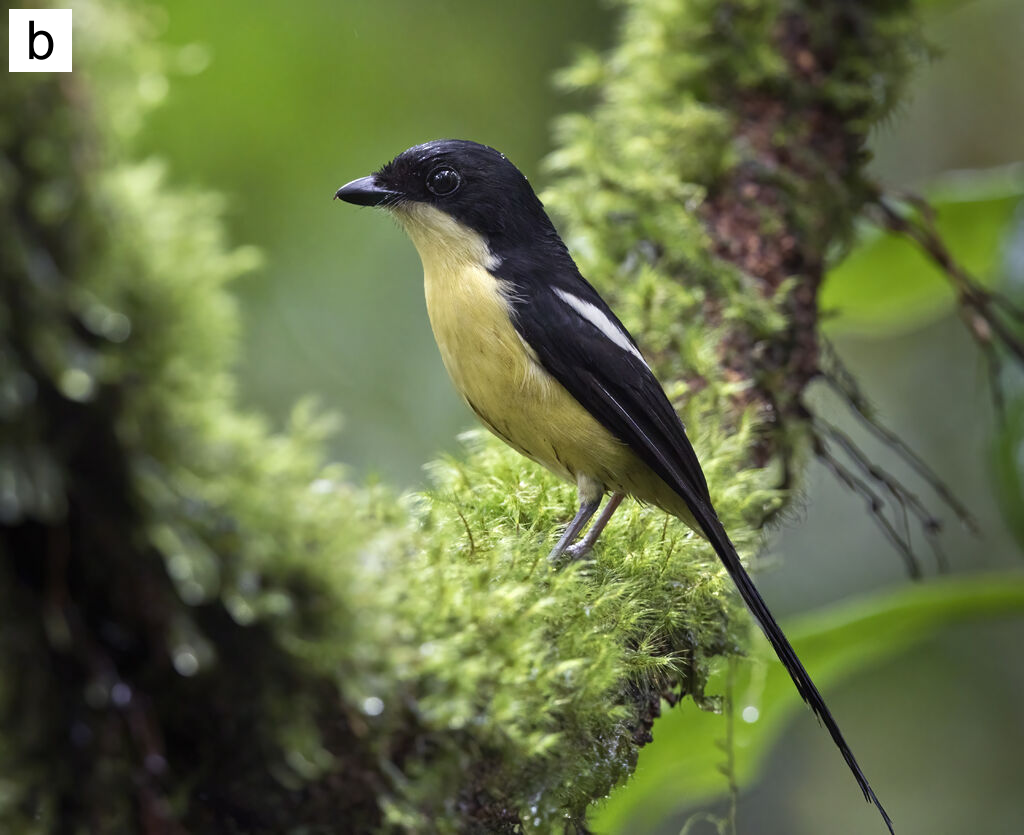
Newton’s Fiscal *Laniusnewtoni*. BIN URI BOLD:AEW1591.

**Figure 2c.**
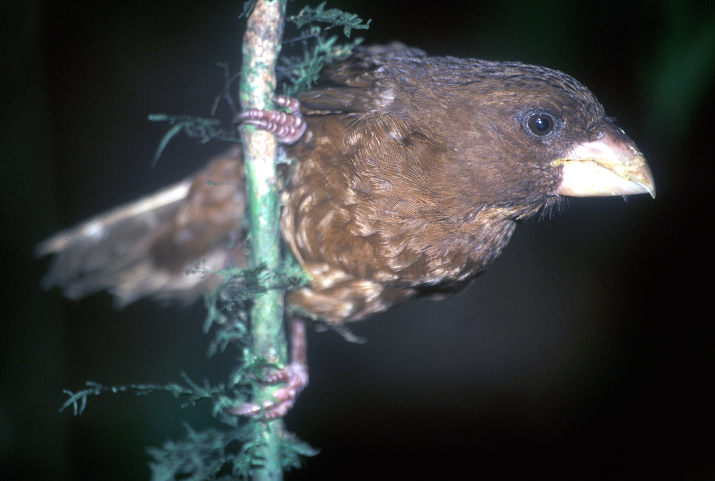
São Tomé Grosbeak *Crithagraconcolor*, the world’s largest canary, BIN URI BOLD:AEV2516.

**Figure 2d.**
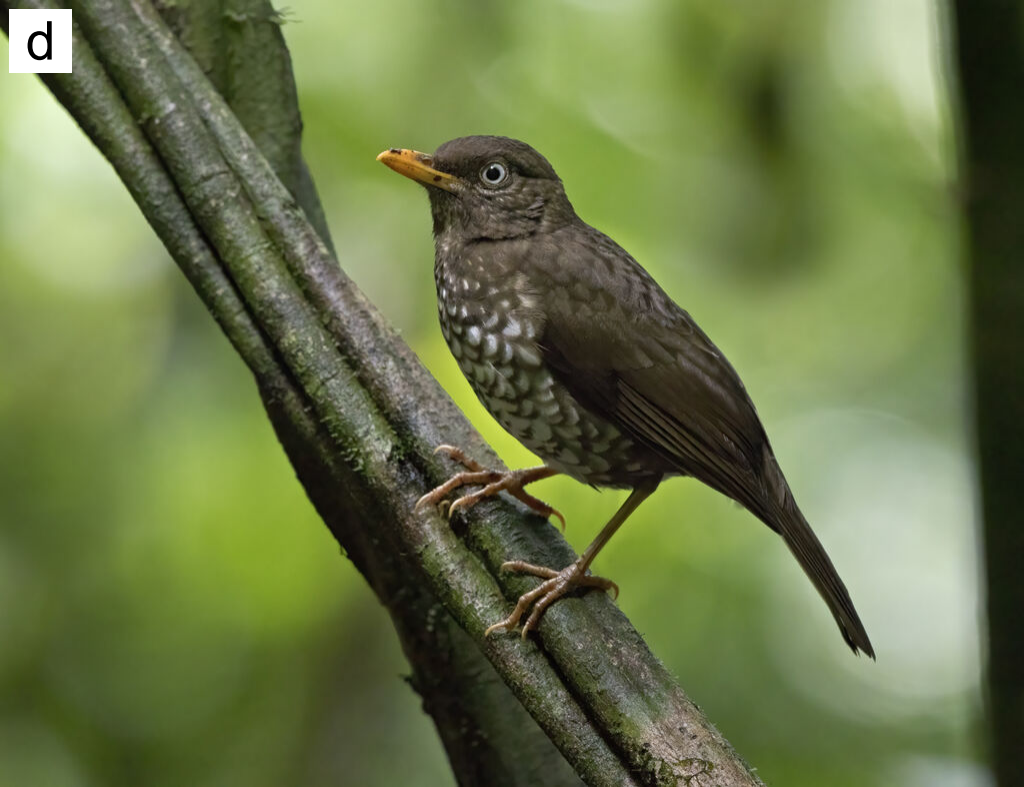
Principe Thrush *Turdusxanthorhynchus*, BIN URI BOLD:AEU9573.

**Figure 2e.**
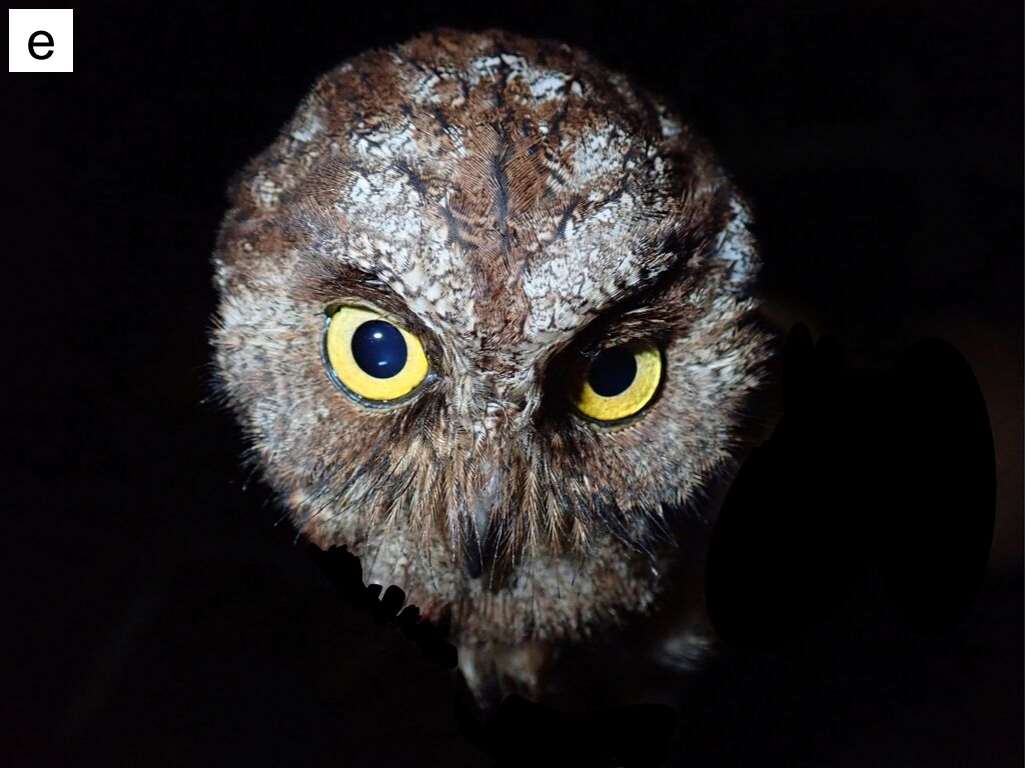
Principe Scops-Owl *Otusbikegila*, BIN URI BOLD:AEV0141.

**Figure 2f.**
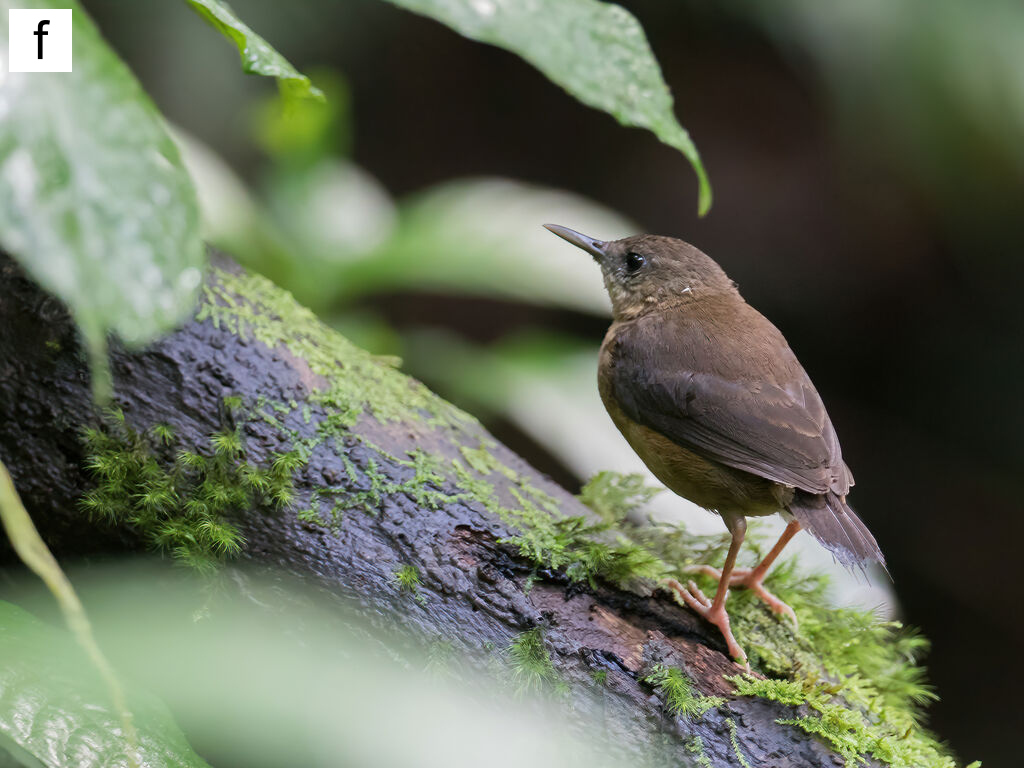
São Tomé Short-tail *Motacillabocagii*. BIN URI BOLD:AEV1800.

**Figure 3a.**
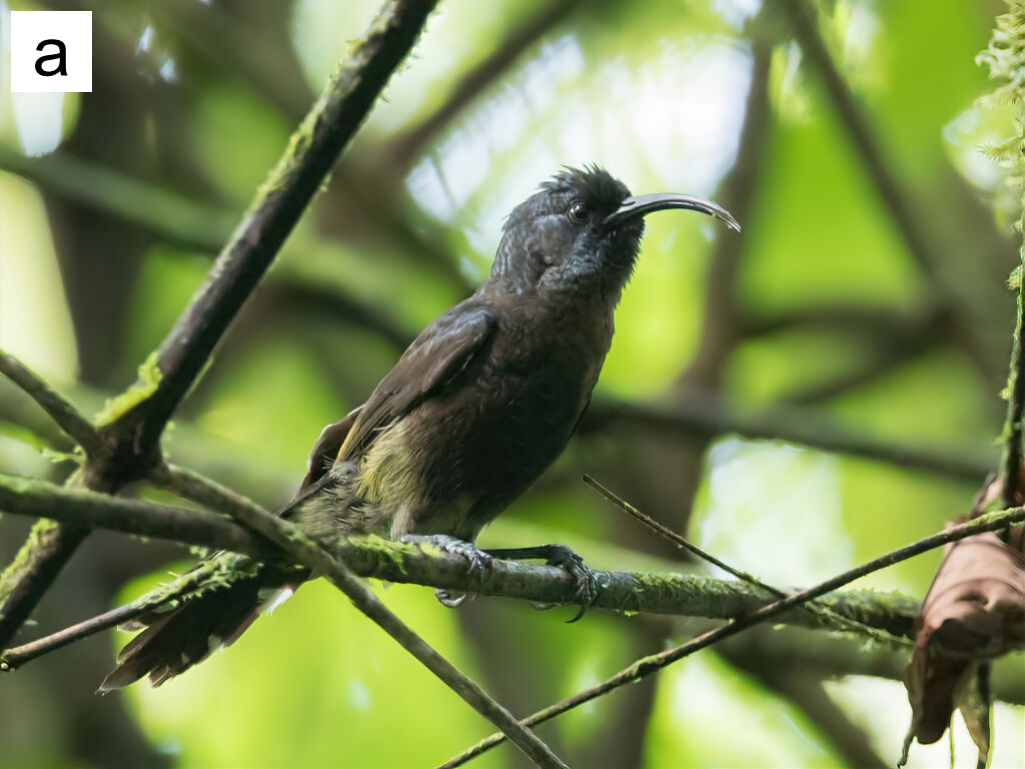
The São Tomé (or Giant) Sunbird *Dreptesthomensis*, BIN URI BOLD:AEV3819 is the world’s largest sunbird and the sister species to the Principe Sunbird *Anabathmishartlaubi*.

**Figure 3b.**
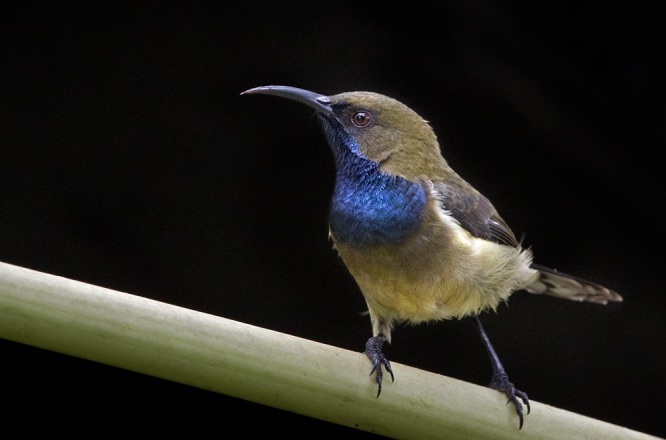
The Principe Sunbird *Anabathmishartlaubi* from Príncipe, BIN URI BOLD:AEW0139.

**Figure 3c.**
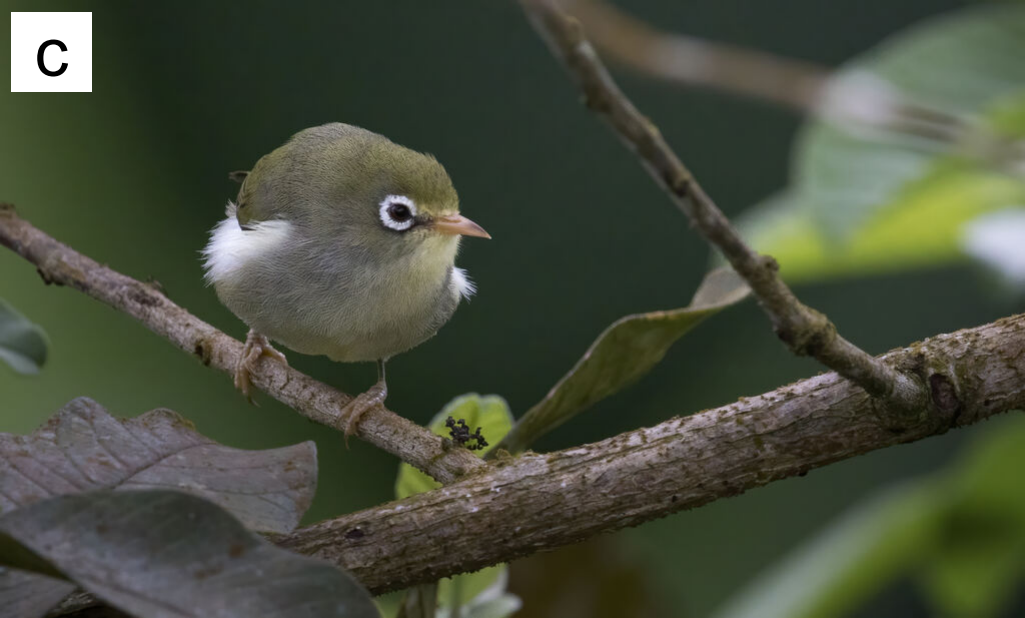
The radiation of Gulf of Guinea white-eyes (Zosteropidae) is modest in species numbers (five species from a single coloniser), but impressive in its diversification rates – one of the highest documented in vertebrates – with extremely fast phenotypic changes affecting some of the species: descendants of the oldest splits, such as the São Tomé White-eye *Zosteropsfeae* BIN URI BOLD:AEW5498, kept the typical white-eye appearance, from which the most recent species diverged.

**Figure 3d.**
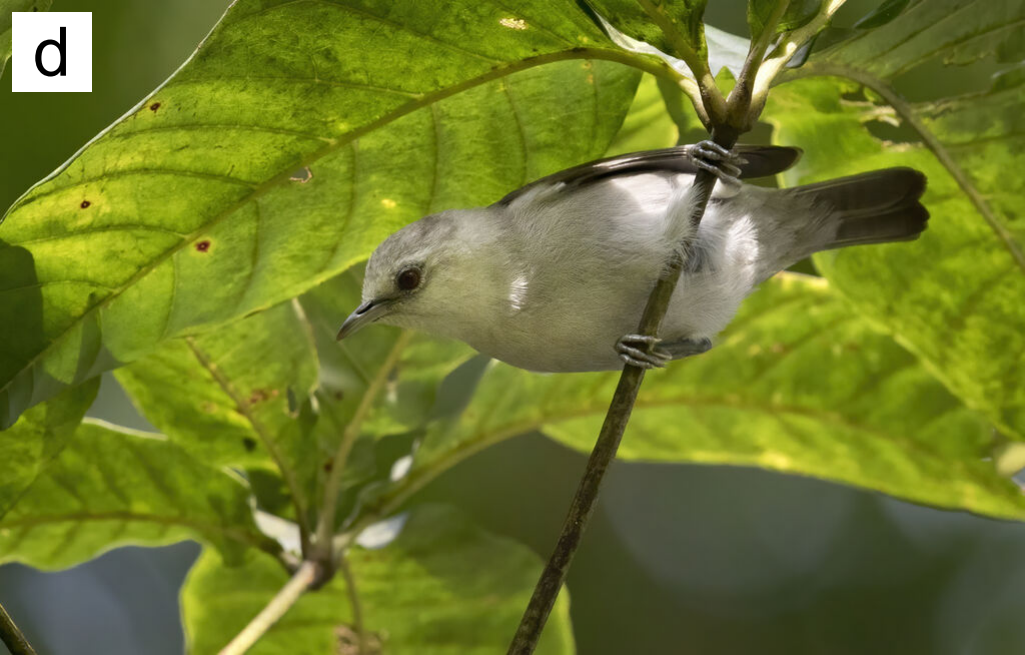
The most recent speciation events of the Gulf of Guinea White-eye radiation, such as the Principe Speirops *Z.leucophaeus* BIN URI BOLD:AEV3242, evolved drastically different phenotypes from the typical white-eye appearance, in a very short time period.

**Figure 3e.**
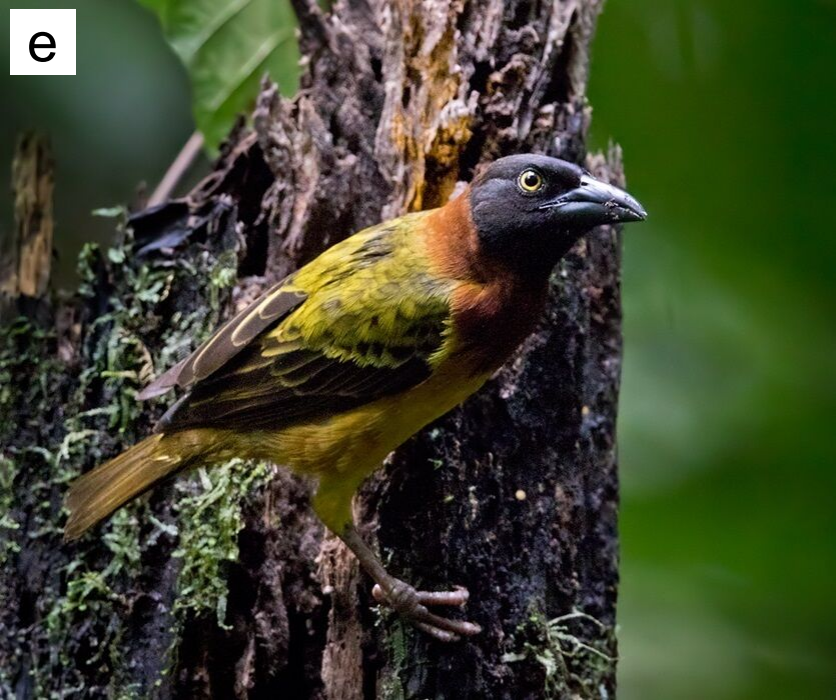
The Giant Weaver *Ploceusgrandis* from São Tomé, the world’s largest weaver, BIN URI BOLD:AEU9687.

**Figure 3f.**
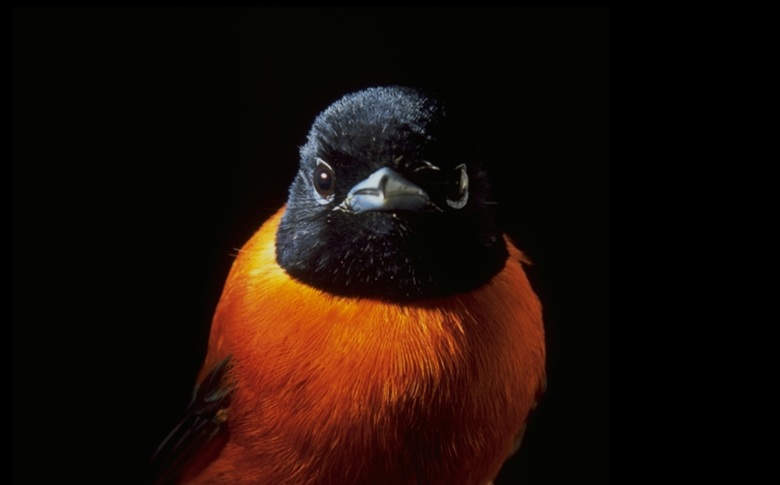
The paradise-flycatcher from Annobón, a taxon in the early stages of – treated by some authorities as an endemic species, the Annobon Paradise-Flycatcher *Terpsiphonesmithii*, but more commonly as an endemic subspecies of the Black-headed Paradise-Flycatcher *T.rufiventersmithii*, BIN URI BOLD:AAV8264.

**Table 1.** List of bird taxa from the oceanic islands of the Gulf of Guinea collected and DNA barcoded within this project. + Indicates endemic species. ++ Indicates endemic subspecies. # Indicate species with new BINs. NA indicates no data. Taxonomy and nomenclature follow de Lima and Melo (2021).

Family	Taxa	DS-IAAST code	BOLD code	BOLD BIN	GenBank accession number
Phasianidae	*Coturnixdelegorgueihistrionica* Hartlaub, 1849++	AV109	IAAST109-22	BOLD:AAR9057	OQ272260
Columbidae	*Columbalarvataprincipalis* (Hartlaub, 1866)++#	AV012	IAAST012-22	BOLD:AEW2890	OQ272236
Columbidae	*Columbalarvataprincipalis* (Hartlaub, 1866)++#	AV013	IAAST013-22	BOLD:AEW2890	OQ272195
Columbidae	*Columbalarvatasimplex* (Hartlaub, 1849)++#	AV065	IAAST065-22	BOLD:AEW2891	OQ272286
Columbidae	*Columbalarvatasimplex* (Hartlaub, 1849)++#	AV066	IAAST066-22	BOLD:AEW2891	OQ272175
Columbidae	*Columbalarvatasimplex* (Hartlaub, 1849)++#	AV067	IAAST067-22	BOLD:AEW2890	OQ272174
Columbidae	*Columbamalherbii* J.Verreaux & E.Verreaux, 1851+#	AV068	IAAST068-22	BOLD:AEW2892	OQ272242
Columbidae	*Columbamalherbii* J.Verreaux & E.Verreaux, 1851+#	AV069	IAAST069-22	BOLD:AEW2892	OQ272194
Columbidae	*Columbamalherbii* J.Verreaux & E.Verreaux, 1851+#	AV070	IAAST070-22	BOLD:AEW2892	OQ272284
Columbidae	*Columbamalherbii* J.Verreaux & E.Verreaux, 1851+#	AV160	IAAST160-22	BOLD:AEW2892	OQ272192
Columbidae	*Columbamalherbii* J.Verreaux & E.Verreaux, 1851+#	AV161	IAAST161-22	BOLD:AEW2892	OQ272244
Columbidae	*Columbathomensis* Barboza du Bocage, 1888+#	AV171	IAAST171-22	BOLD:AEV8749	OQ272235
Columbidae	*Treronsanctithomae* (J.F.Gmelin, 1789)+	AV073	IAAST073-22	NA	OQ272148
Cuculidae	Chrysococcyxcupreusinsularum Moreau & Chapin, 1951++#	AV075	IAAST075-22	BOLD:AEV6409	OQ272205
Cuculidae	*Chrysococcyxcupreusinsularum* Moreau & Chapin, 1951++#	AV076	IAAST076-22	BOLD:AEV6409	OQ272222
Apodidae	*Apusaffinisbannermani* Hartert, 1928++	AV007	IAAST007-22	BOLD:AAJ8912	OQ272159
Apodidae	*Apusaffinisbannermani* Hartert, 1928++	AV008	IAAST008-22	BOLD:AAJ8912	OQ272254
Apodidae	*Apusaffinisbannermani* Hartert, 1928++	AV009	IAAST009-22	BOLD:AAJ8912	OQ272261
Apodidae	*Cypsiurusparvusbrachypterus* (Reichenow, 1903)#	AV058	IAAST058-22	BOLD:AEU7484	OQ272272
Apodidae	*Zoonavenathomensis* (Hartert, 1900)+#	AV059	IAAST059-22	BOLD:AEV2307	OQ272266
Apodidae	*Zoonavenathomensis* (Hartert, 1900)+#	AV060	IAAST060-22	BOLD:AEV2307	OQ272285
Rallidae	*Paragallinulaangulata* (Sundevall, 1851)	AV135	IAAST135-22	BOLD:ACS4051	OQ272152
Ardeidae	*Bubulcusibis* (Linnaeus, 1758)	AV010	IAAST010-22	BOLD:AAB5991	OQ272249
Ardeidae	*Bubulcusibis* (Linnaeus, 1758)	AV011	IAAST011-22	BOLD:AAB5991	OQ272193
Threskiornithidae	*Bostrychiabocagei* (Chapin, 1923)+#	AV157	IAAST157-22	BOLD:AEW0523	OQ272214
Threskiornithidae	*Bostrychiabocagei* (Chapin, 1923)+#	AV158	IAAST158-22	BOLD:AEW0523	OQ272181
Strigidae	*Otusbikegila* Melo et al., 2022+#	AV037	IAAST037-22	BOLD:AEV0141	OQ272203
Strigidae	*Otusbikegila* Melo et al., 2022+#	AV038	IAAST038-22	BOLD:AEV0141	OQ272191
Strigidae	*Otusbikegila* Melo et al., 2022+#	AV039	IAAST039-22	BOLD:AEV0141	OQ272209
Strigidae	*Otushartlaubi* (Giebel, 1872)+#	AV136	IAAST136-22	BOLD:AEW5101	OQ272206
Strigidae	*Otushartlaubi* (Giebel, 1872)+#	AV137	IAAST137-22	BOLD:AEW5101	OQ272185
Strigidae	*Otushartlaubi* (Giebel, 1872)+#	AV138	IAAST138-22	BOLD:AEW5101	OQ272230
Strigidae	*Otussenegalensisfeae* (Salvadori, 1903)++#	AV156	IAAST156-22	BOLD:AEW5100	OQ272267
Alcedinidae	*Corythorniscristatusnais* (Kaup, 1848)++	AV001	IAAST001-22	BOLD:ACO5243	OQ272183
Alcedinidae	*Corythorniscristatusnais* (Kaup, 1848)++	AV002	IAAST002-22	BOLD:ACO5243	OQ272208
Alcedinidae	*Corythorniscristatusnais* (Kaup, 1848)++	AV003	IAAST003-22	BOLD:ACO5243	OQ272246
Alcedinidae	*Corythorniscristatusthomensis* Salvadori, 1902++	AV055	IAAST055-22	BOLD:ACO5243	OQ272255
Alcedinidae	*Corythorniscristatusthomensis* Salvadori, 1902++	AV056	IAAST056-22	BOLD:ACO5243	OQ272278
Alcedinidae	*Corythorniscristatusthomensis* Salvadori, 1902++	AV057	IAAST057-22	BOLD:ACO5243	OQ272283
Alcedinidae	*Halcyonmalimbicadryas* Hartlaub, 1854++	AV004	IAAST004-22	BOLD:ABA9399	OQ272263
Alcedinidae	*Halcyonmalimbicadryas* Hartlaub, 1854++	AV005	IAAST005-22	BOLD:ABA9399	OQ272293
Alcedinidae	*Halcyonmalimbicadryas* Hartlaub, 1854++	AV006	IAAST006-22	BOLD:ABA9399	OQ272169
Psittaculidae	*Agapornispullariuspullarius* (Linnaeus, 1758)#	AV134	IAAST134-22	BOLD:AEU9050	OQ272221
Oriolidae	*Orioluscrassirostris* Hartlaub, 1857+#	AV106	IAAST106-22	BOLD:AEU8006	OQ272232
Oriolidae	*Orioluscrassirostris* Hartlaub, 1857+#	AV107	IAAST107-22	BOLD:AEU8006	OQ272141
Oriolidae	*Orioluscrassirostris* Hartlaub, 1857+#	AV108	IAAST108-22	BOLD:AEU8006	OQ272162
Monarchidae	*Terpsiphoneatrochalybeia* (Thomson, 1842)+#	AV094	IAAST094-22	BOLD:AEU7438	OQ272167
Monarchidae	*Terpsiphoneatrochalybeia* (Thomson, 1842)+#	AV095	IAAST095-22	BOLD:AEU7438	OQ272146
Monarchidae	*Terpsiphoneatrochalybeia* (Thomson, 1842)+#	AV096	IAAST096-22	BOLD:AEU7438	OQ272223
Monarchidae	*Terpsiphonerufiventersmithii* (Fraser, 1843)++	AV151	IAAST151-22	BOLD:AAV8264	OQ272197
Monarchidae	*Terpsiphonerufiventersmithii* (Fraser, 1843)++	AV152	IAAST152-22	BOLD:AAV8264	OQ272165
Laniidae	*Laniusnewtoni* Bocage, 1891+#	AV091	IAAST091-22	BOLD:AEW1591	OQ272157
Laniidae	*Laniusnewtoni* Bocage, 1891+#	AV092	IAAST092-22	BOLD:AEW1591	OQ272178
Laniidae	*Laniusnewtoni* Bocage, 1891+#	AV093	IAAST093-22	BOLD:AEW1591	OQ272250
Cisticolidae	*Priniamolleri* Bocage, 1887+#	AV062	IAAST062-22	BOLD:AEW0581	OQ272188
Cisticolidae	*Priniamolleri* Bocage, 1887+#	AV063	IAAST063-22	BOLD:AEW0581	OQ272161
Cisticolidae	*Priniamolleri* Bocage, 1887+#	AV064	IAAST064-22	BOLD:AEW0581	OQ272166
Sylviidae	*Sylviacommuniscommunis* Latham, 1787	AV044	IAAST044-22	BOLD:AAB8930	OQ272155
Sylviidae	*Sylviacommuniscommunis* Latham, 1787	AV045	IAAST045-22	BOLD:AAB8930	OQ272274
Sylviidae	*Sylviadohrni* (Hartlaub, 1866)+#	AV047	IAAST047-22	BOLD:AEV0164	OQ272292
Zosteropidae	*Zosteropsfeae* Salvadori, 1901+#	AV145	IAAST145-22	BOLD:AEW5498	OQ272279
Zosteropidae	*Zosteropsfeae* Salvadori, 1901+#	AV146	IAAST146-22	BOLD:AEW5498	OQ272156
Zosteropidae	*Zosteropsfeae* Salvadori, 1901+#	AV147	IAAST147-22	BOLD:AEW5498	OQ272150
Zosteropidae	*Zosteropsficedulinus* Hartlaub, 1866+#	AV167	IAAST167-22	BOLD:AEW5499	OQ272186
Zosteropidae	*Zosteropsgriseovirescens* Bocage, 1893+#	AV153	IAAST153-22	BOLD:AEV3243	OQ272234
Zosteropidae	*Zosteropsgriseovirescens* Bocage, 1893+#	AV154	IAAST154-22	BOLD:AEV3243	OQ272151
Zosteropidae	*Zosteropsgriseovirescens* Bocage, 1893+#	AV155	IAAST155-22	BOLD:AEV3243	OQ272154
Zosteropidae	*Zosteropsleucophaeus* (Hartlaub, 1857)+#	AV052	IAAST052-22	BOLD:AEV3242	OQ272269
Zosteropidae	*Zosteropsleucophaeus* (Hartlaub, 1857)+#	AV053	IAAST053-22	BOLD:AEV3242	OQ272256
Zosteropidae	*Zosteropsleucophaeus* (Hartlaub, 1857)+#	AV054	IAAST054-22	BOLD:AEV3242	OQ272231
Zosteropidae	*Zosteropslugubris* Hartlaub, 1848+#	AV148	IAAST148-22	BOLD:AEV3242	OQ272228
Zosteropidae	*Zosteropslugubris* Hartlaub, 1848+#	AV149	IAAST149-22	BOLD:AEV3242	OQ272202
Zosteropidae	*Zosteropslugubris* Hartlaub, 1848+#	AV150	IAAST150-22	BOLD:AEV3242	OQ272224
Sturnidae	*Lamprotornisornatus* (Daudin, 1800)+	AV040	IAAST040-22	BOLD:AAX0614	OQ272145
Sturnidae	*Lamprotornisornatus* (Daudin, 1800)+	AV041	IAAST041-22	BOLD:AAX0614	OQ272163
Sturnidae	*Lamprotornisornatus* (Daudin, 1800)+	AV042	IAAST042-22	BOLD:AAX0614	OQ272211
Sturnidae	*Lamprotornissplendidussplendidus* (Vieillot, 1822)	AV043	IAAST043-22	BOLD:AAX0614	OQ272281
Sturnidae	*Onychognathusfulgidusfulgidus* Hartlaub, 1849++#	AV139	IAAST139-22	BOLD:AEV6478	OQ272252
Sturnidae	*Onychognathusfulgidusfulgidus* Hartlaub, 1849++#	AV140	IAAST140-22	BOLD:AEV6478	OQ272271
Sturnidae	*Onychognathusfulgidusfulgidus* Hartlaub, 1849++#	AV141	IAAST141-22	BOLD:AEV6478	OQ272258
Turdidae	*Turdusolivaceofuscus* Hartlaub, 1852+#	AV142	IAAST142-22	BOLD:AEV0477	OQ272187
Turdidae	*Turdusolivaceofuscus* Hartlaub, 1852+#	AV143	IAAST143-22	BOLD:AEV0477	OQ272248
Turdidae	*Turdusolivaceofuscus* Hartlaub, 1852+#	AV144	IAAST144-22	BOLD:AEV0477	OQ272245
Turdidae	*Turdusxanthorhynchus* Salvadori, 1901+#	AV049	IAAST049-22	BOLD:AEU9573	OQ272276
Turdidae	*Turdusxanthorhynchus* Salvadori, 1901+#	AV050	IAAST050-22	BOLD:AEU9573	OQ272218
Turdidae	*Turdusxanthorhynchus* Salvadori, 1901+#	AV051	IAAST051-22	BOLD:AEU9573	OQ272275
Nectariniidae	*Anabathmishartlaubii* (Hartlaub, 1857)+#	AV028	IAAST028-22	BOLD:AEW0139	OQ272173
Nectariniidae	*Anabathmishartlaubii* (Hartlaub, 1857)+#	AV029	IAAST029-22	BOLD:AEW0139	OQ272237
Nectariniidae	*Anabathmishartlaubii* (Hartlaub, 1857)+#	AV030	IAAST030-22	BOLD:AEW0139	OQ272212
Nectariniidae	*Anabathmisnewtonii* (Bocage, 1887)+#	AV100	IAAST100-22	BOLD:AEW0138	OQ272270
Nectariniidae	*Anabathmisnewtonii* (Bocage, 1887)+#	AV101	IAAST101-22	BOLD:AEW0138	OQ272176
Nectariniidae	*Anabathmisnewtonii* (Bocage, 1887)+#	AV102	IAAST102-22	BOLD:AEW0138	OQ272288
Nectariniidae	*Cyanomitraolivaceaobscura* (Jardine, 1842)	AV031	IAAST031-22	BOLD:AAS2298	OQ272216
Nectariniidae	*Cyanomitraolivaceaobscura* (Jardine, 1842)	AV032	IAAST032-22	BOLD:AAS2298	OQ272171
Nectariniidae	*Cyanomitraolivaceaobscura* (Jardine, 1842)	AV033	IAAST033-22	BOLD:AAS2298	OQ272290
Nectariniidae	*Dreptesthomensis* (Bocage, 1889)+#	AV103	IAAST103-22	BOLD:AEV3819	OQ272240
Nectariniidae	*Dreptesthomensis* (Bocage, 1889)+#	AV104	IAAST104-22	BOLD:AEV3819	OQ272294
Nectariniidae	*Dreptesthomensis* (Bocage, 1889)+#	AV105	IAAST105-22	BOLD:AEV3819	OQ272168
Ploceidae	*Euplectesalbonotatusasymmetrurus* (Reichenow, 1891)#	AV110	IAAST110-22	BOLD:AEW3408	OQ272239
Ploceidae	*Euplectesalbonotatusasymmetrurus* (Reichenow, 1891)#	AV111	IAAST111-22	BOLD:AEW3408	OQ272201
Ploceidae	*Euplectesalbonotatusasymmetrurus* (Reichenow, 1891)#	AV112	IAAST112-22	BOLD:AEW3408	OQ272253
Ploceidae	*Euplectesaureus* (Gmelin, 1789)#	AV113	IAAST113-22	BOLD:AEW3409	OQ272225
Ploceidae	*Euplectesaureus* (Gmelin, 1789)#	AV114	IAAST114-22	BOLD:AEW3409	OQ272247
Ploceidae	*Euplectesaureus* (Gmelin, 1789)#	AV115	IAAST115-22	BOLD:AEW3409	OQ272287
Ploceidae	*Euplecteshordeaceushordeaceus* (Linnaeus, 1758)	AV116	IAAST116-22	BOLD:ABA9525	OQ272164
Ploceidae	*Euplecteshordeaceushordeaceus* (Linnaeus, 1758)	AV118	IAAST118-22	BOLD:ABA9525	OQ272204
Ploceidae	*Ploceuscucullatusnigriceps* (E.L.Layard, 1867)#	AV119	IAAST119-22	BOLD:AEV2326	OQ272273
Ploceidae	*Ploceuscucullatusnigriceps* (E.L.Layard, 1867)#	AV120	IAAST120-22	BOLD:AEV2326	OQ272227
Ploceidae	*Ploceuscucullatusnigriceps* (E.L.Layard, 1867)#	AV121	IAAST121-22	BOLD:AEV2326	OQ272251
Ploceidae	*Ploceusgrandis* (G.R.Gray, 1844)+#	AV122	IAAST122-22	BOLD:AEU9687	OQ272264
Ploceidae	*Ploceusgrandis* (G.R.Gray, 1844)+#	AV123	IAAST123-22	BOLD:AEU9687	OQ272207
Ploceidae	*Ploceusgrandis* (G.R.Gray, 1844)+#	AV124	IAAST124-22	BOLD:AEU9687	OQ272179
Ploceidae	*Ploceusprinceps* (Bonaparte, 1850)+#	AV034	IAAST034-22	BOLD:AEU9686	OQ272177
Ploceidae	*Ploceusprinceps* (Bonaparte, 1850)+#	AV035	IAAST035-22	BOLD:AEU9686	OQ272257
Ploceidae	*Ploceusprinceps* (Bonaparte, 1850)+#	AV036	IAAST036-22	BOLD:AEU9686	OQ272160
Ploceidae	*Ploceussanctithomae* (Hartlaub, 1848)+#	AV125	IAAST125-22	BOLD:AEV3313	OQ272280
Ploceidae	*Ploceussanctithomae* (Hartlaub, 1848)+#	AV126	IAAST126-22	BOLD:AEV3313	OQ272184
Ploceidae	*Ploceussanctithomae* (Hartlaub, 1848)+#	AV127	IAAST127-22	BOLD:AEV3313	OQ272226
Ploceidae	*Ploceusvelatus* Vieillot, 1819	AV128	IAAST128-22	BOLD:ACA3852	OQ272170
Ploceidae	*Ploceusvelatus* Vieillot, 1819	AV129	IAAST129-22	BOLD:ACA3852	OQ272291
Ploceidae	*Ploceusvelatus* Vieillot, 1819	AV130	IAAST130-22	BOLD:ACA3852	OQ272200
Ploceidae	*Queleaerythrops* (Hartlaub, 1848)	AV131	IAAST131-22	BOLD:ABA9905	OQ272295
Ploceidae	*Queleaerythrops* (Hartlaub, 1848)	AV132	IAAST132-22	BOLD:ABA9905	OQ272210
Ploceidae	*Queleaerythrops* (Hartlaub, 1848)	AV133	IAAST133-22	BOLD:ABA9905	OQ272262
Estrildidae	*Estrildaastrildjagoensis* Alexander, 1898	AV015	IAAST015-22	BOLD:AAU4407	OQ272140
Estrildidae	*Estrildaastrildjagoensis* Alexander, 1898	AV016	IAAST016-22	BOLD:AAU4407	OQ272144
Estrildidae	*Estrildaastrildjagoensis* Alexander, 1898	AV017	IAAST017-22	BOLD:AAU4407	OQ272189
Estrildidae	*Estrildaastrildjagoensis* Alexander, 1898	AV077	IAAST077-22	BOLD:AAU4407	OQ272199
Estrildidae	*Estrildaastrildjagoensis* Alexander, 1898	AV079	IAAST079-22	BOLD:AAU4407	OQ272158
Estrildidae	*Nigritabicolorbrunnescens* Reichenow, 1902	AV021	IAAST021-22	BOLD:AAZ3356	OQ272277
Estrildidae	*Spermestescucullatacucullata* Swainson, 1837	AV018	IAAST018-22	BOLD:ABA9398	OQ272229
Estrildidae	*Spermestescucullatacucullata* Swainson, 1837	AV019	IAAST019-22	BOLD:ABA9398	OQ272172
Estrildidae	*Spermestescucullatacucullata* Swainson, 1837	AV020	IAAST020-22	BOLD:ABA9398	OQ272259
Estrildidae	*Spermestescucullatacucullata* Swainson, 1837	AV080	IAAST080-22	BOLD:ABA9398	OQ272233
Estrildidae	*Spermestescucullatacucullata* Swainson, 1837	AV081	IAAST081-22	BOLD:ABA9398	OQ272289
Estrildidae	*Spermestescucullatacucullata* Swainson, 1837	AV082	IAAST082-22	BOLD:ABA9398	OQ272180
Estrildidae	*Uraeginthusangolensisangolensis* (Linnaeus, 1758)	AV083	IAAST083-22	BOLD:AAN3755	OQ272241
Estrildidae	*Uraeginthusangolensisangolensis* (Linnaeus, 1758)	AV085	IAAST085-22	BOLD:AAN3755	OQ272220
Motacillidae	*Motacillabocagii* (Sharpe, 1892)+#	AV097	IAAST097-22	BOLD:AEV1800	OQ272213
Motacillidae	*Motacillabocagii* (Sharpe, 1892)+#	AV098	IAAST098-22	BOLD:AEV1800	OQ272149
Motacillidae	*Motacillabocagii* (Sharpe, 1892)+#	AV099	IAAST099-22	BOLD:AEV1800	OQ272282
Fringillidae	*Crithagraconcolor* (Barboza du Bocage, 1888)+#	AV086	IAAST086-22	BOLD:AEV2516	OQ272238
Fringillidae	*Crithagraconcolor* (Barboza du Bocage, 1888)+#	AV087	IAAST087-22	BOLD:AEV2516	OQ272147
Fringillidae	*Crithagramozambicatando* (W.L.Sclater & Mackworth-Praed, 1918)#	AV172	IAAST172-22	BOLD:AEU6526	OQ272217
Fringillidae	*Crithagramozambicatando* (W.L.Sclater & Mackworth-Praed, 1918)#	AV173	IAAST173-22	BOLD:AEU6526	OQ272215
Fringillidae	*Crithagramozambicatando* (W.L.Sclater & Mackworth-Praed, 1918)#	AV174	IAAST174-22	BOLD:AEU6526	OQ272219
Fringillidae	*Crithagrarufobrunneafradei* (Naurois, 1975)++#	AV022	IAAST022-22	BOLD:AEV2517	OQ272142
Fringillidae	*Crithagrarufobrunneafradei* (Naurois, 1975)++#	AV023	IAAST023-22	BOLD:AEV2517	OQ272143
Fringillidae	*Crithagrarufobrunneafradei* (Naurois, 1975)++#	AV024	IAAST024-22	BOLD:AEV2517	OQ272196
Fringillidae	*Crithagrarufobrunnearufobrunnea* (G.R.Gray, 1862)++#	AV025	IAAST025-22	BOLD:AEV2517	OQ272243
Fringillidae	*Crithagrarufobrunnearufobrunnea* (G.R.Gray, 1862)++#	AV026	IAAST026-22	BOLD:AEV2517	OQ272153
Fringillidae	*Crithagrarufobrunnearufobrunnea* (G.R.Gray, 1862)++#	AV027	IAAST027-22	BOLD:AEV2517	OQ272190
Fringillidae	*Crithagrarufobrunneathomensis* (Barboza du Bocage, 1888)++#	AV088	IAAST088-22	BOLD:AEV2516	OQ272265
Fringillidae	*Crithagrarufobrunneathomensis* (Barboza du Bocage, 1888)++#	AV089	IAAST089-22	BOLD:AEV2516	OQ272182
Fringillidae	*Crithagrarufobrunneathomensis* (Barboza du Bocage, 1888)++#	AV090	IAAST090-22	BOLD:AEV2516	OQ272268
